# Supplementary material for: Metagenomic, metabolomic, and lipidomic shifts associated with fecal microbiota transplantation for recurrent Clostridioides difficile infection
Source: mSphere. 2024 Oct 8;9(10):e00706-24. doi: 10.1128/msphere.00706-24 (PMC11520286; doi:10.1128/msphere.00706-24)
Supplement: Supplemental figures — Figures S1-S6. [file msphere.00706-24-s0001.pdf]

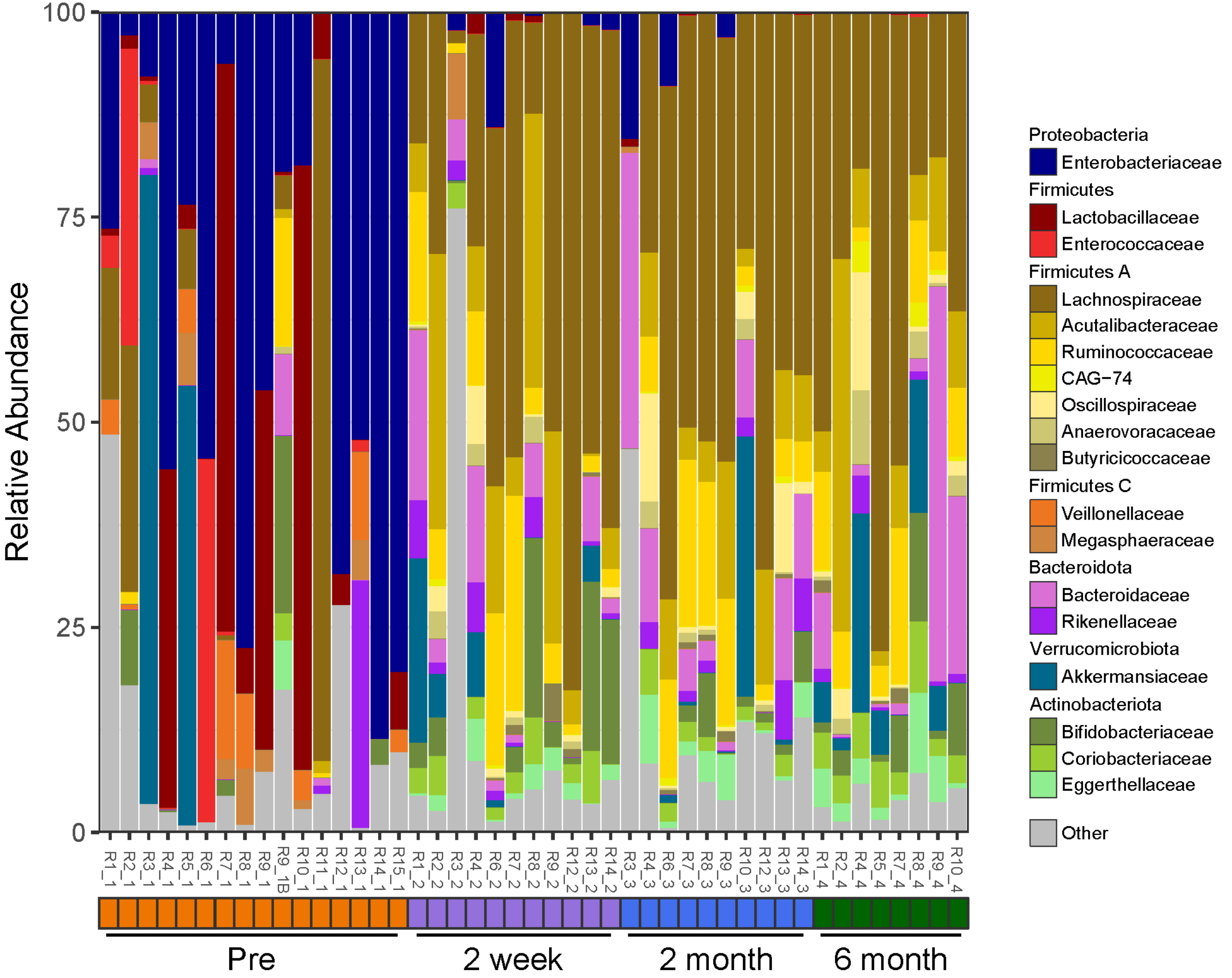

Figure S1. Relative abundance of bacterial Family membership changes between pre- and post-FMT. Relative abundance of bacterial Family membership for each sample pre- and post-FMT samples. Fecal samples were collected pre-FMT (n=16: orange), and then 2 week (n=11: purple), 2 month (n=10: blue), and 6 month (n=8: green) post-FMT for a total of (n=29) total post-FMT samples.

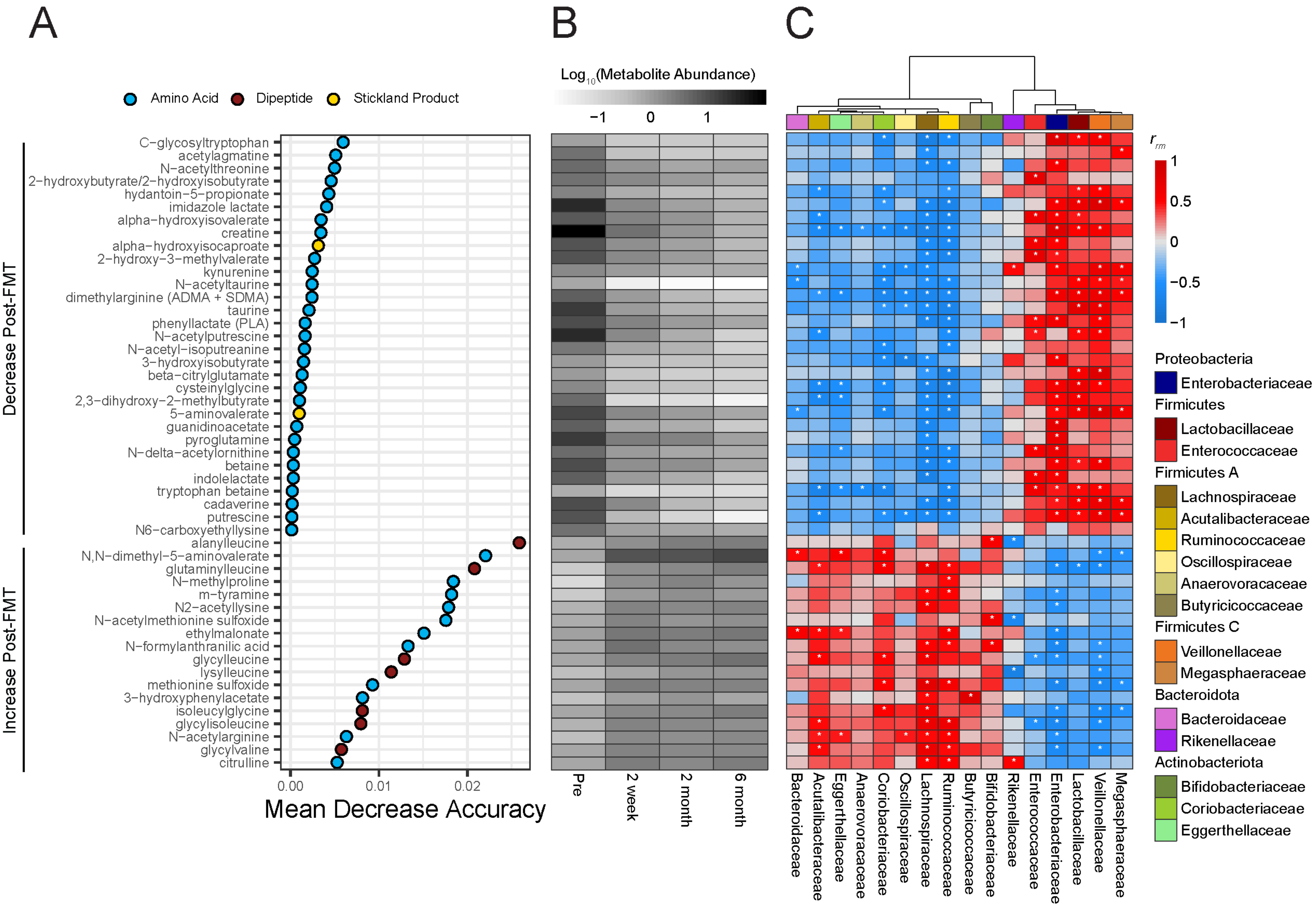

Figure S2. Changes in amino acids and dipeptides pre- and post-FMT. (A) RFA of amino acids and dipeptides defined by untargeted metabolomics in stool samples from patients undergoing FMT for rCDI. Amino acids and dipeptides were categorized into those that decrease after FMT or increase after FMT. All amino acids and dipeptides listed were observed to be significant between time points or after FMT by linear mixed models with Bonferroni correction ($q \leq 0.05$) (Table S2). The OOB error rate for RFA in determining pre- vs post-FMT using these metabolites is 13.33%. **(B)** Mean of log 10 transformation of counts per million (CPM) + 1 of reads assigned to each species as labelled in A. **(C)** Repeated measure correlation between abundance of important amino acids.

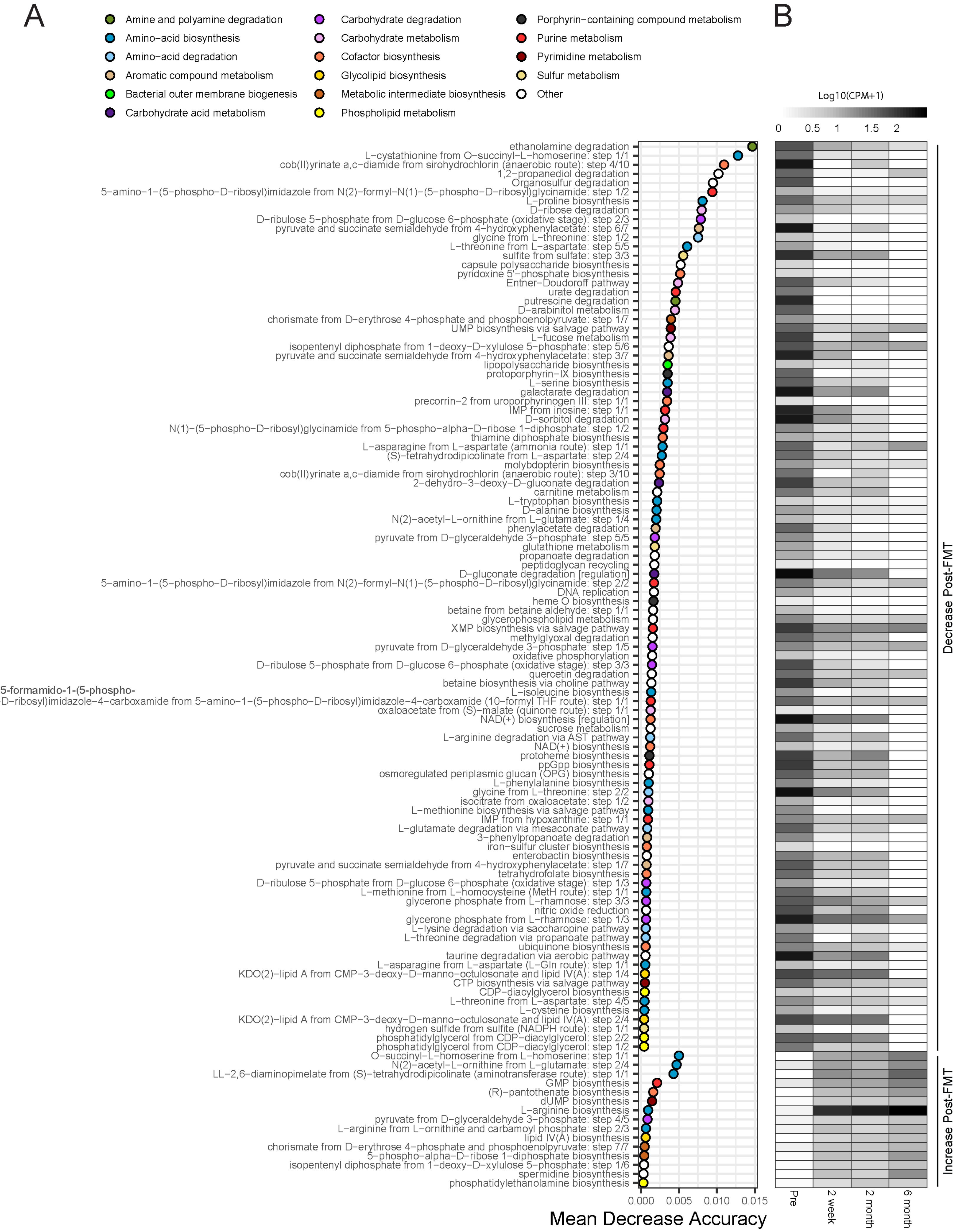

Figure S3. Microbial pathways identified through metagenomics change in response to FMT. (A) RFA of microbial pathways belonging to the labelled UniPathway defined by metagenomics in stool samples from patients undergoing FMT for rCDI. Microbial pathways were categorized into those that decrease after FMT or increase after FMT. All microbial pathways listed were observed to be significant between time points or after FMT by linear mixed models with Bonferroni correction (q ≤ 0.05) (Table S2). The OOB error rate for RFA in determining pre- vs post-FMT using these metabolites is 8.89%. **(B)** Mean of log 10 transformation of counts per million (CPM) + 1 of reads assigned to each species as labelled in A.

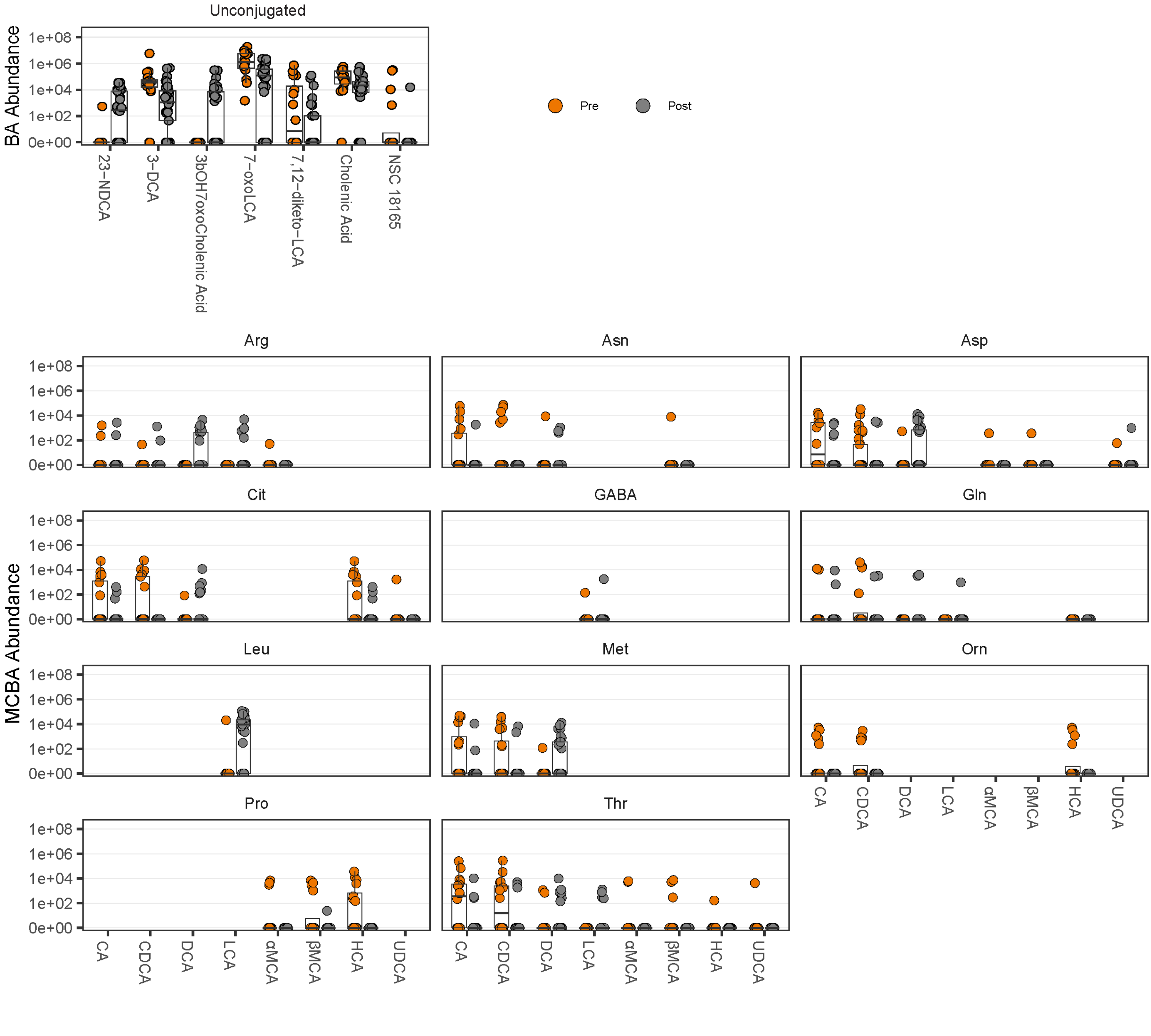

Figure S4. MCBA abundance changes between pre- and post-FMT. MCBA and unconjugated bile acid abundance for each amino acid/sterol core combination not found to be significant between time points or after FMT by linear mixed models. Box plots depict IQR with whiskers depicting the most extreme value or 1.5x IQR, whichever is lower.

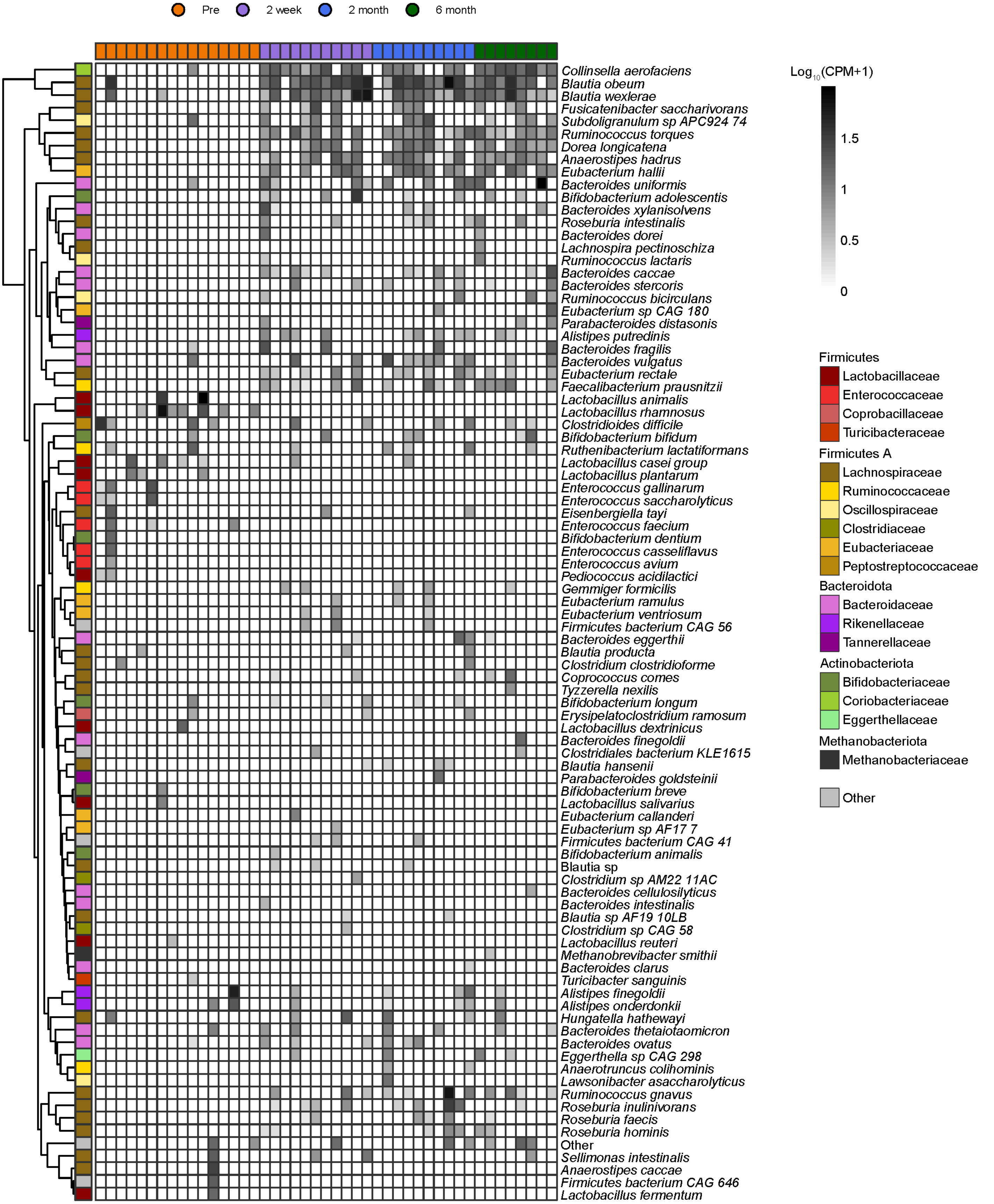

Figure S5. Varying contributions by species to BSH abundance in FMT samples. Heatmap of the sum of BSH genes encoded by each species transformed by $\log_{10}$(CPM+1). Rows were clustered by using complete linkage clustering with Euclidian distances of total BSH abundance attributed to each species.

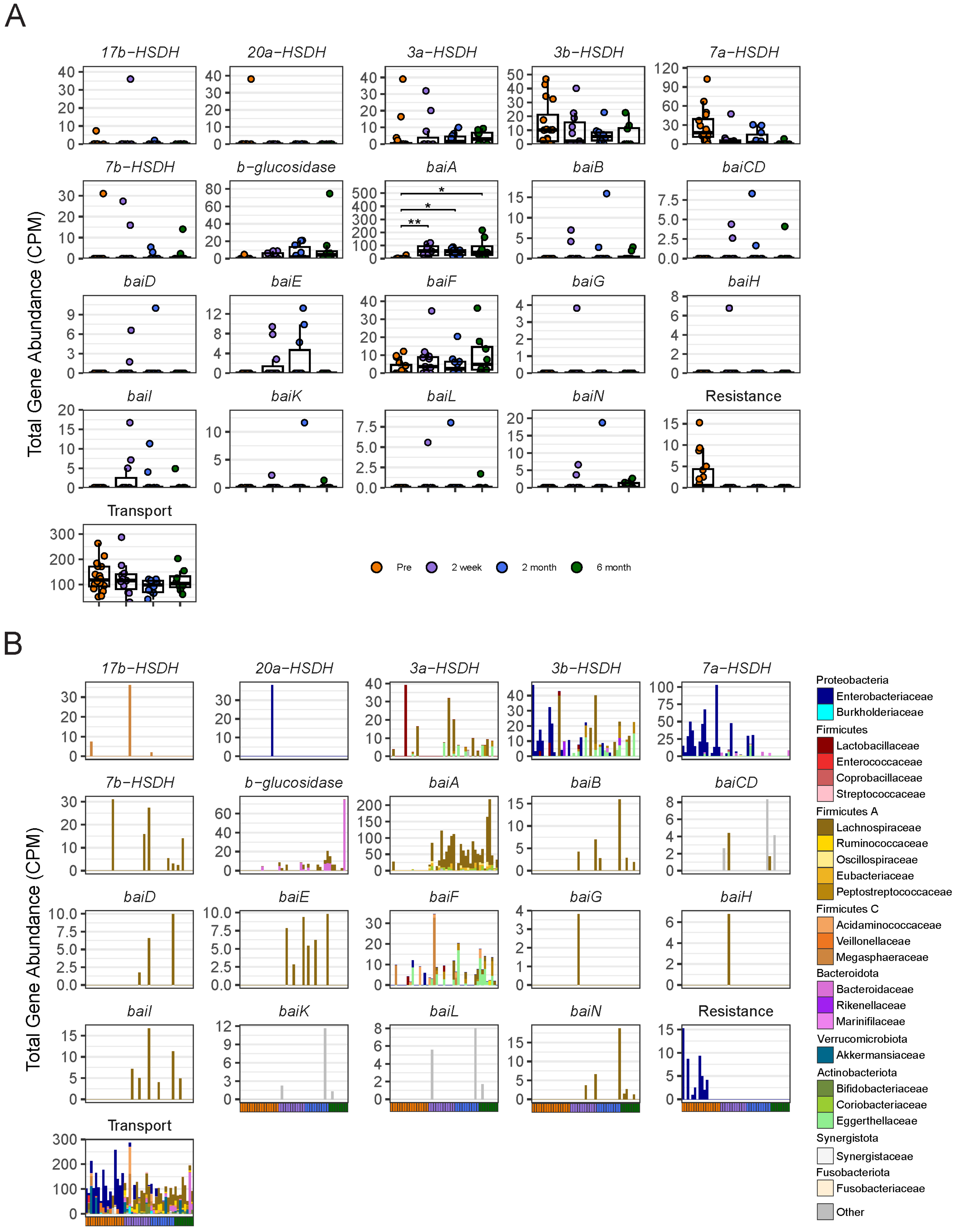

Figure S6. Abundance and bacterial Family level contributions of other bile acid altering genes change in response to FMT. (A) Counts per million (CPM) of bile acid related genes identified via shallow shotgun metagenomics. **(B)** Stacked bar plot of bile acid related genes split by the Family that encode it for each stool sample. Genes categorized as “Other” were identified via BLASTx and do not have species information.
