# Supplementary material for: Metagenomic, metabolomic, and lipidomic shifts associated with fecal microbiota transplantation for recurrent Clostridioides difficile infection
Source: mSphere. 2024 Oct 8;9(10):e00706-24. doi: 10.1128/msphere.00706-24 (PMC11520286; doi:10.1128/msphere.00706-24)
Supplement: Supplemental material — Supplemental table captions. [file msphere.00706-24-s0002.docx]

# Supplemental Material

# Supplemental information:

Table S1. Linear Models

Table S2. All significant metabolites

Table S3. Genes identified

Table S4. Lipidomics standards and LC settings
